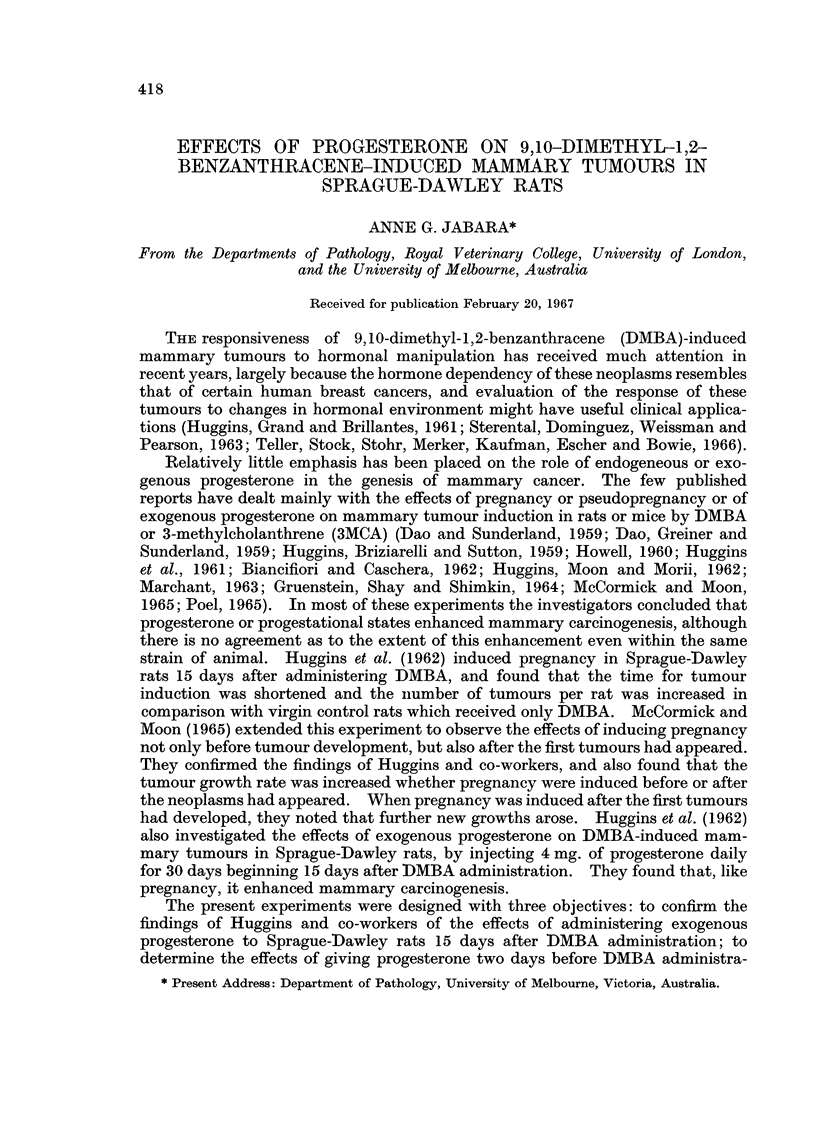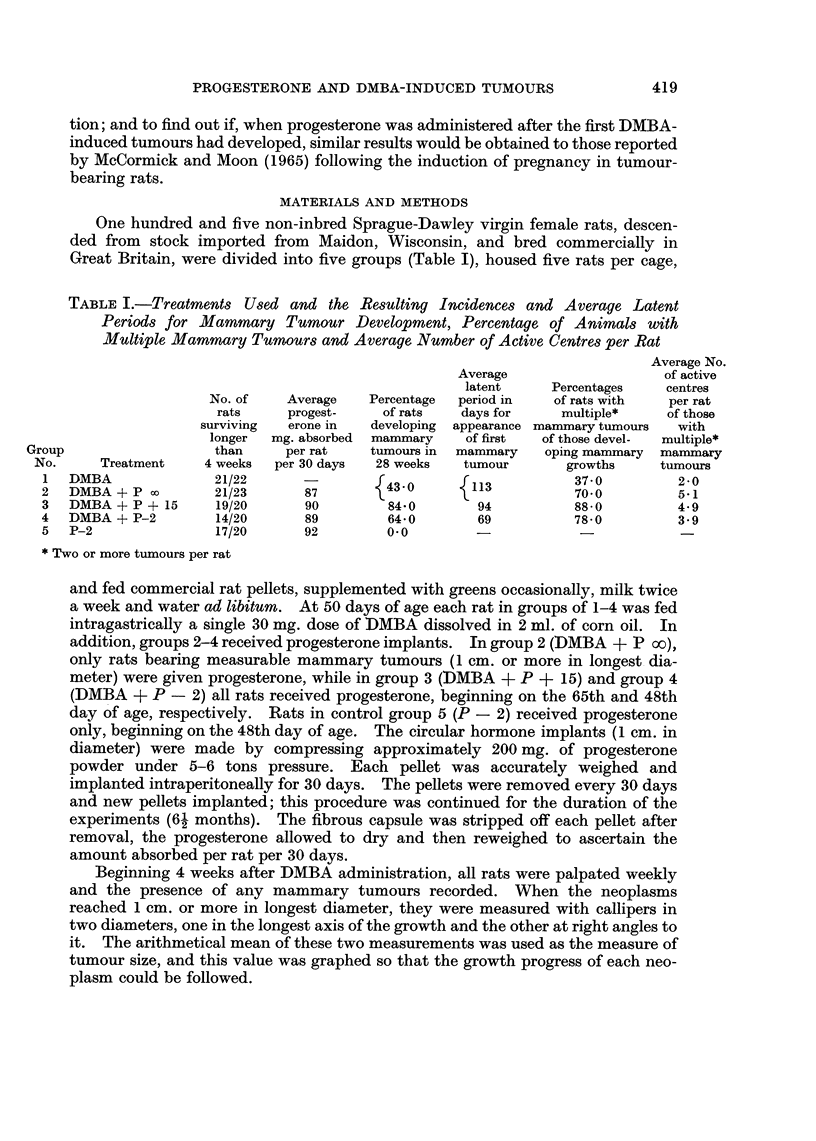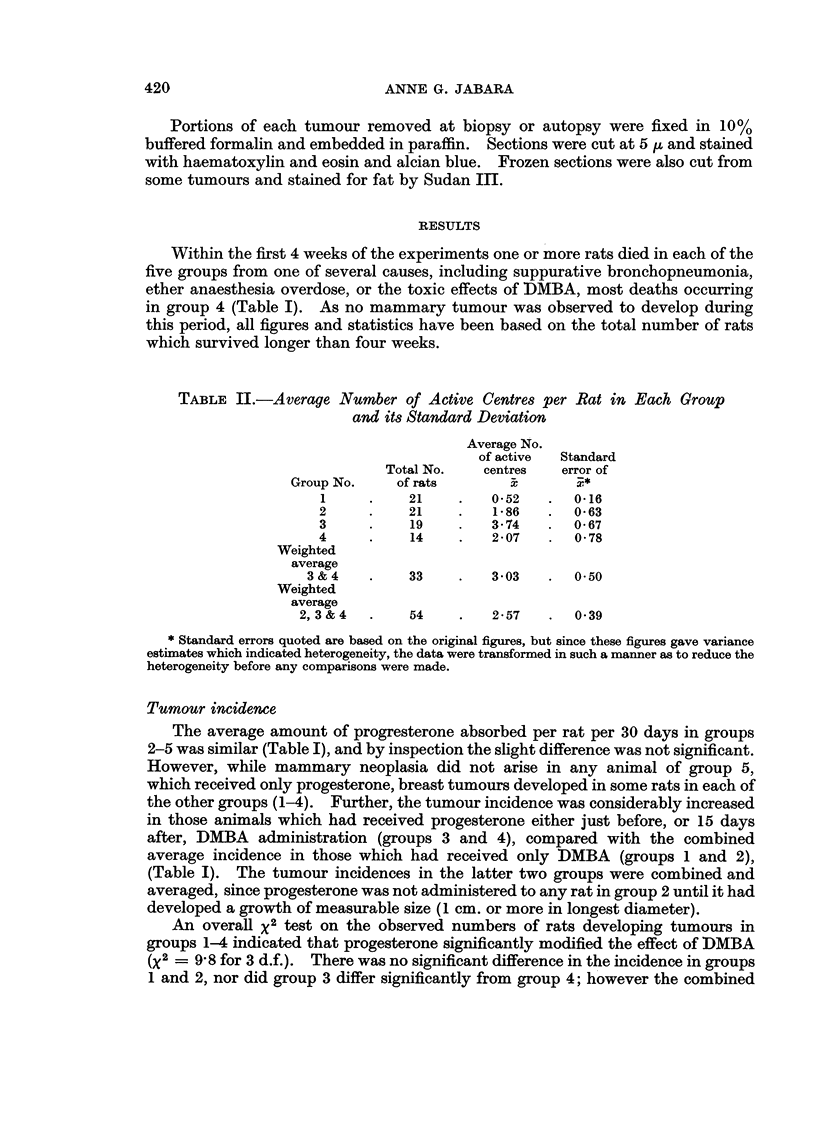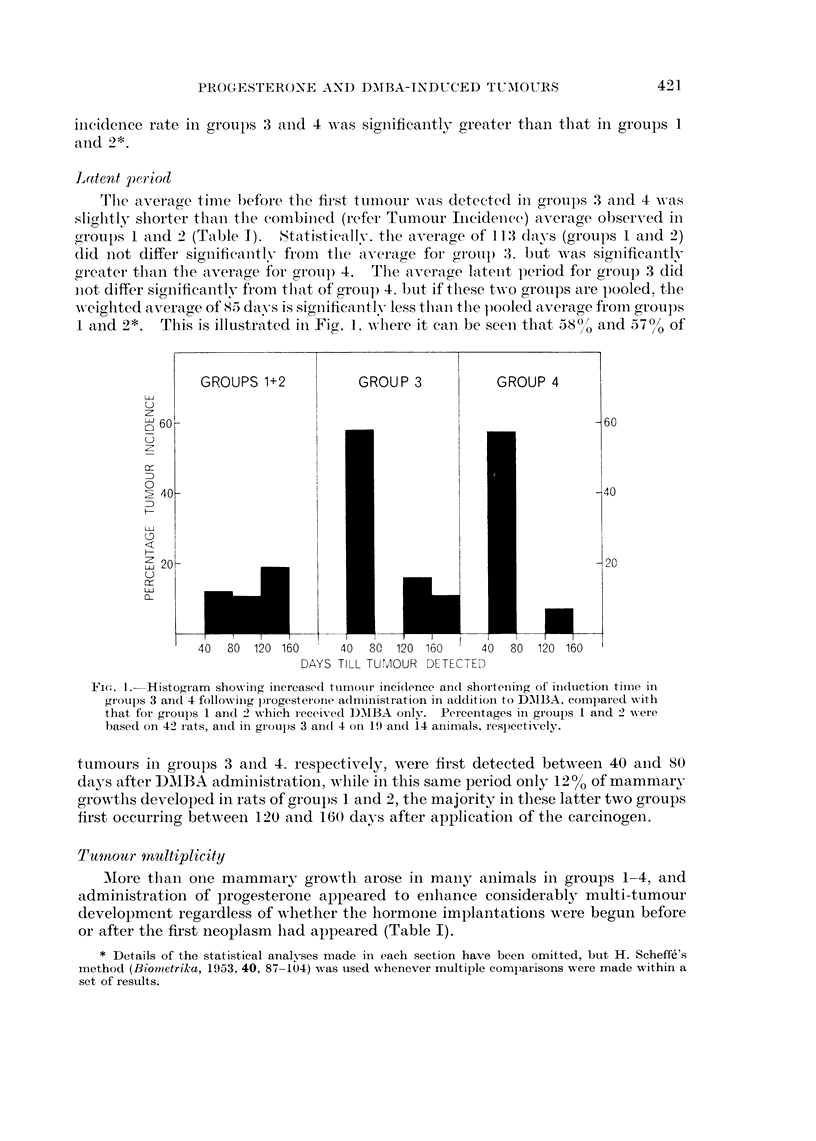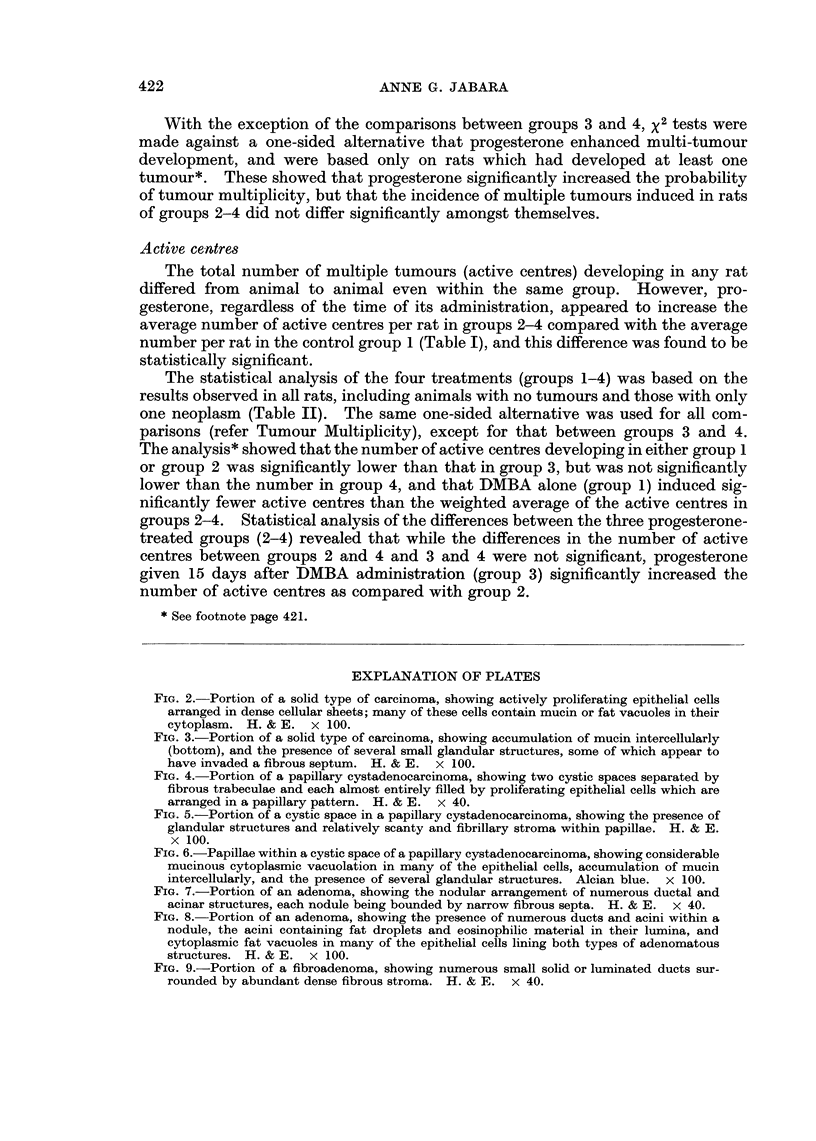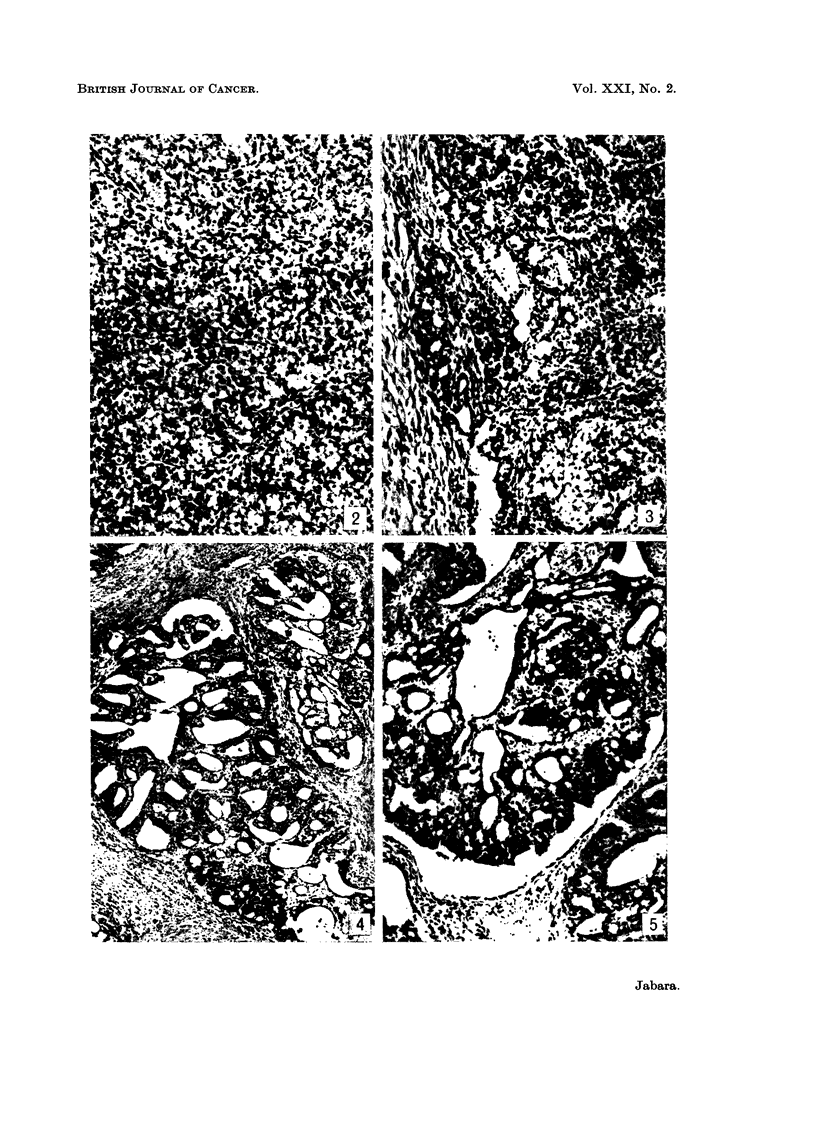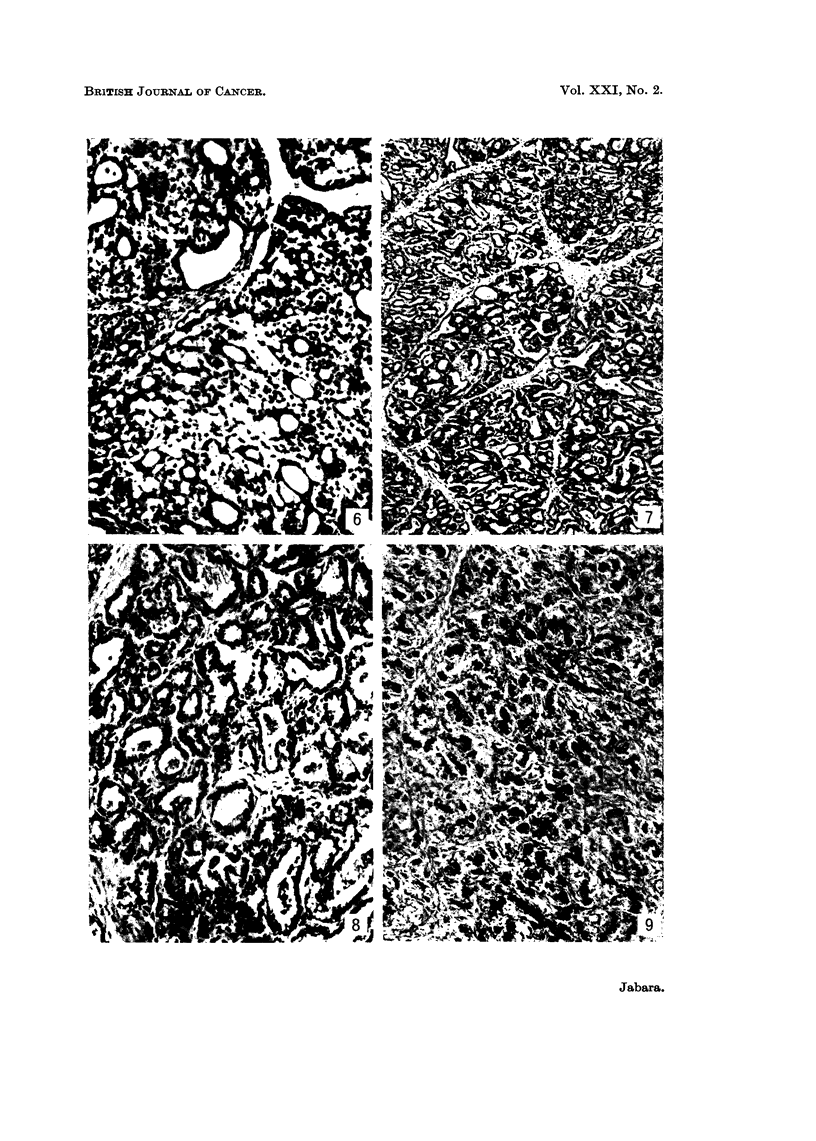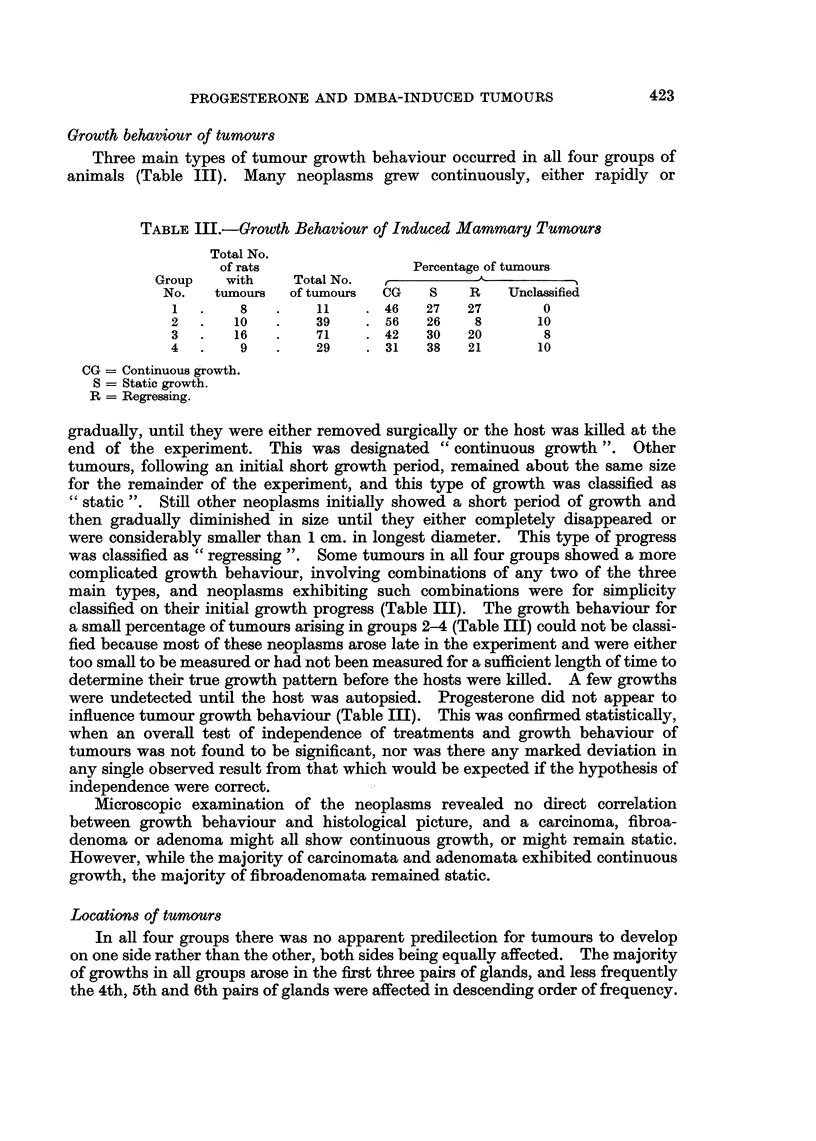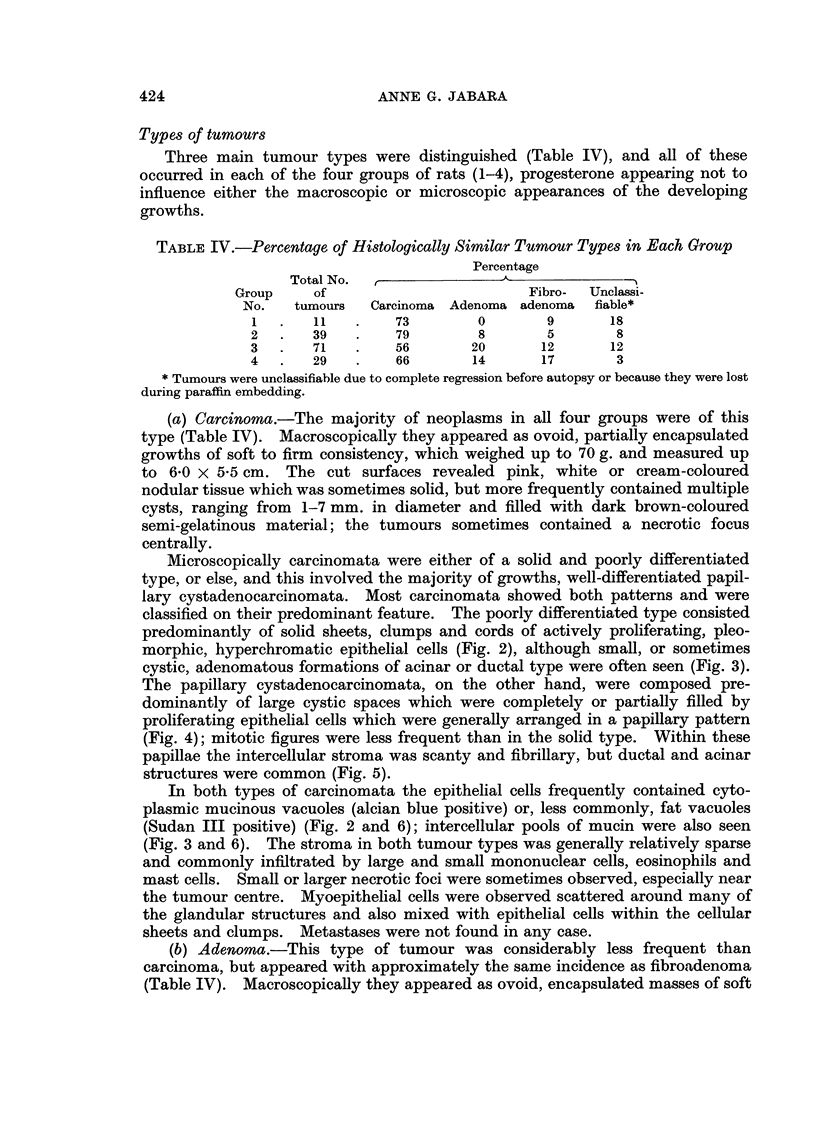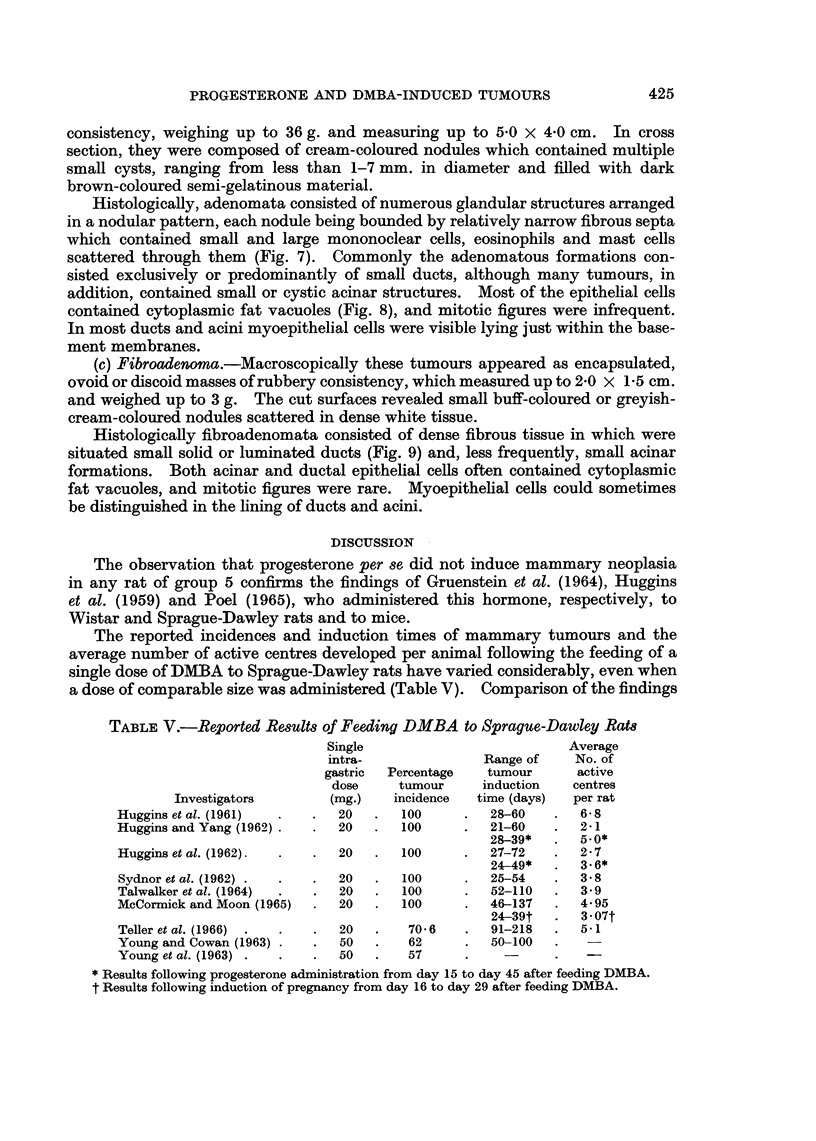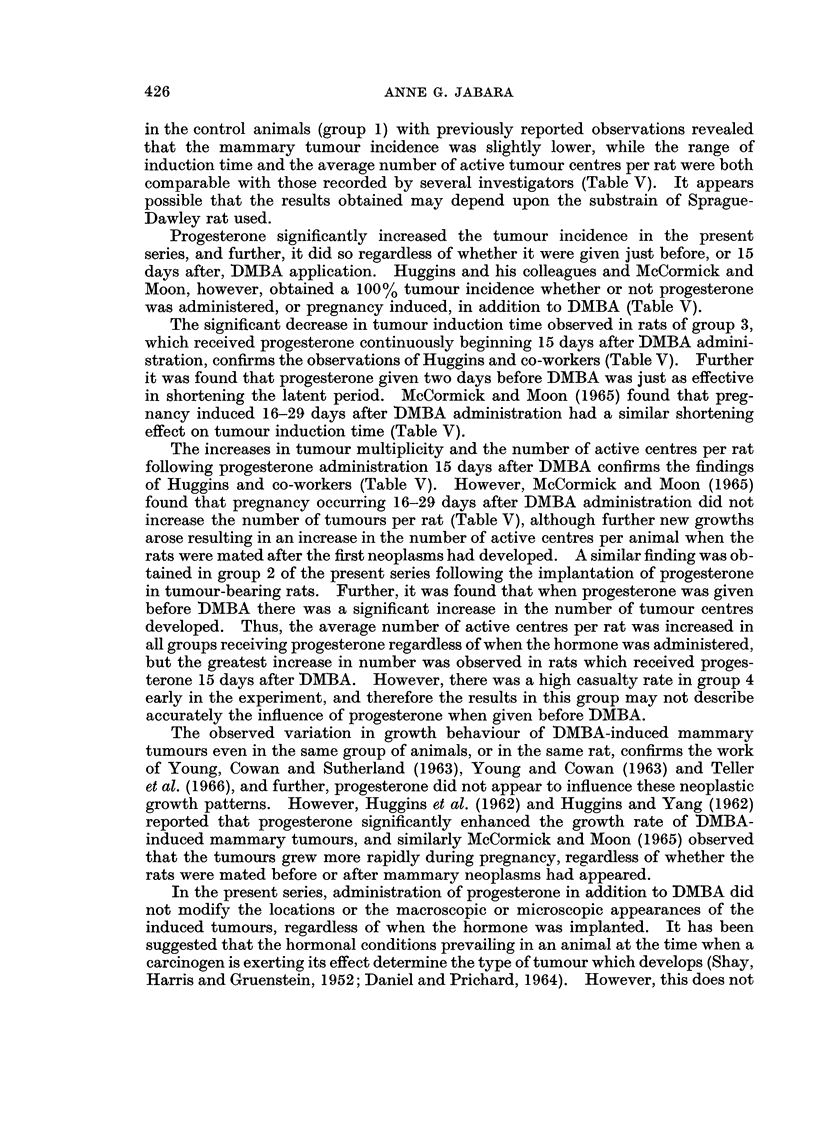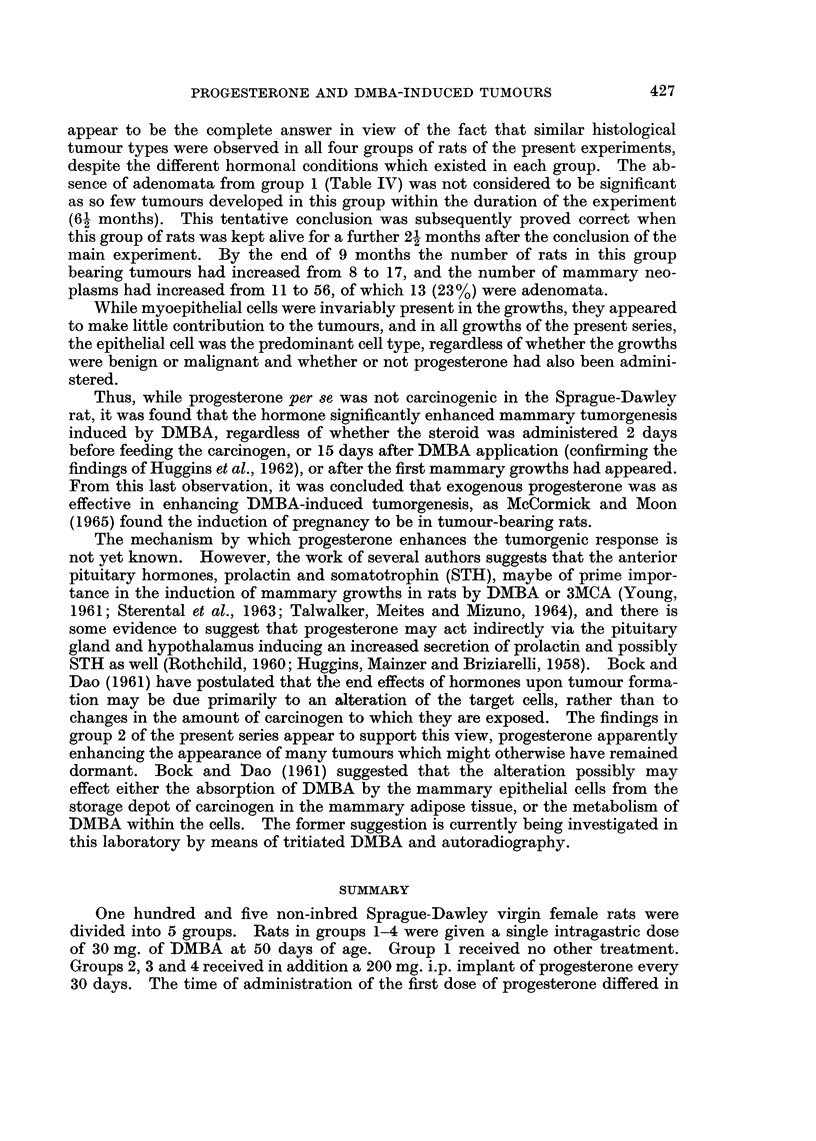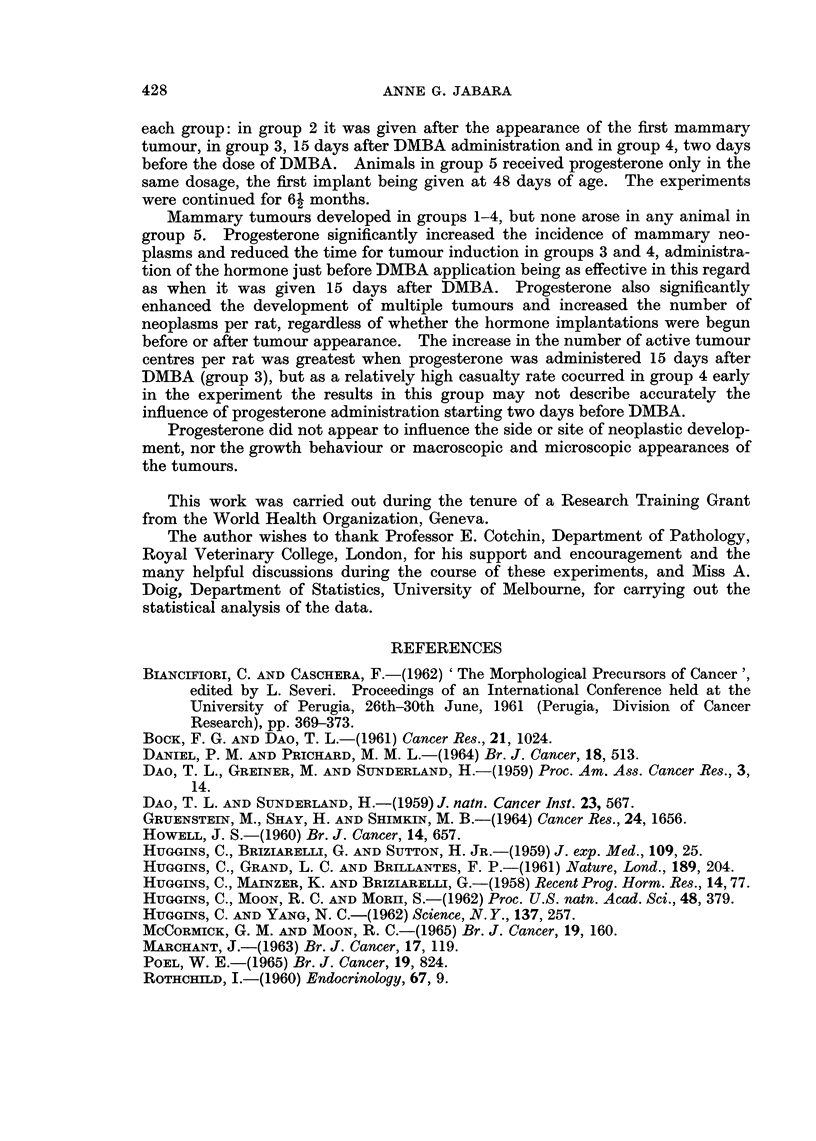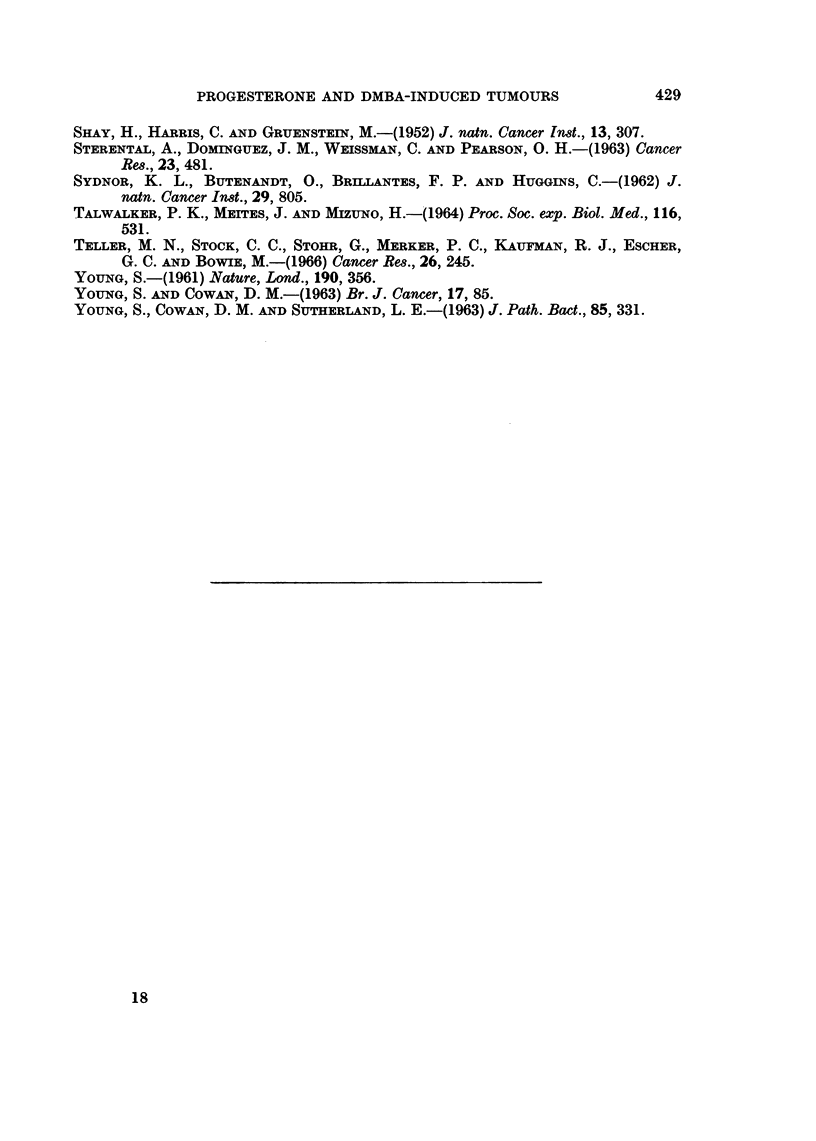# Effects of progesterone on 9,10-dimethyl-1,2-benzanthracene-induced mammary tumours in Sprague-Dawley rats.

**DOI:** 10.1038/bjc.1967.48

**Published:** 1967-06

**Authors:** A. G. Jabara

## Abstract

**Images:**


### EFFECTS OF PROGESTERONE ON 9,10-DIMETHYL-1,2-BENZANTHRACENE-INDUCED MAMMARY TUMOURS IN SPRAGUE-DAWLEY RATS

ANNE G. JABARA*

*From the Departments of Pathology, Royal Veterinary College, University of London, and the University of Melbourne, Australia*

Received for publication February 20, 1967

THE responsiveness of 9,10-dimethyl-1,2-benzanthracene (DMBA)-induced mammary tumours to hormonal manipulation has received much attention in recent years, largely because the hormone dependency of these neoplasms resembles that of certain human breast cancers, and evaluation of the response of these tumours to changes in hormonal environment might have useful clinical applications (Huggins, Grand and Brillantes, 1961; Sterental, Dominguez, Weissman and Pearson, 1963; Teller, Stock, Stohr, Merker, Kaufman, Escher and Bowie, 1966).

Relatively little emphasis has been placed on the role of endogeneous or exogenous progesterone in the genesis of mammary cancer. The few published reports have dealt mainly with the effects of pregnancy or pseudopregnancy or of exogenous progesterone on mammary tumour induction in rats or mice by DMBA or 3-methylcholanthrene (3MCA) (Dao and Sunderland, 1959; Dao, Greiner and Sunderland, 1959; Huggins, Briziarelli and Sutton, 1959; Howell, 1960; Huggins *et al.*, 1961; Biancifiori and Caschera, 1962; Huggins, Moon and Morii, 1962; Marchant, 1963; Gruenstein, Shay and Shimkin, 1964; McCormick and Moon, 1965; Poel, 1965). In most of these experiments the investigators concluded that progesterone or progestational states enhanced mammary carcinogenesis, although there is no agreement as to the extent of this enhancement even within the same strain of animal. Huggins *et al.* (1962) induced pregnancy in Sprague-Dawley rats 15 days after administering DMBA, and found that the time for tumour induction was shortened and the number of tumours per rat was increased in comparison with virgin control rats which received only DMBA. McCormick and Moon (1965) extended this experiment to observe the effects of inducing pregnancy not only before tumour development, but also after the first tumours had appeared. They confirmed the findings of Huggins and co-workers, and also found that the tumour growth rate was increased whether pregnancy were induced before or after the neoplasms had appeared. When pregnancy was induced after the first tumours had developed, they noted that further new growths arose. Huggins *et al.* (1962) also investigated the effects of exogenous progesterone on DMBA-induced mammary tumours in Sprague-Dawley rats, by injecting 4 mg. of progesterone daily for 30 days beginning 15 days after DMBA administration. They found that, like pregnancy, it enhanced mammary carcinogenesis.

The present experiments were designed with three objectives: to confirm the findings of Huggins and co-workers of the effects of administering exogenous progesterone to Sprague-Dawley rats 15 days after DMBA administration; to determine the effects of giving progesterone two days before DMBA administra-

* Present Address: Department of Pathology, University of Melbourne, Victoria, Australia.

tion; and to find out if, when progesterone was administered after the first DMBA-induced tumours had developed, similar results would be obtained to those reported by McCormick and Moon (1965) following the induction of pregnancy in tumour-bearing rats.

### MATERIALS AND METHODS

One hundred and five non-inbred Sprague-Dawley virgin female rats, descended from stock imported from Maidon, Wisconsin, and bred commercially in Great Britain, were divided into five groups (Table I), housed five rats per cage,

TABLE I.—*Treatments Used and the Resulting Incidences and Average Latent Periods for Mammary Tumour Development, Percentage of Animals with Multiple Mammary Tumours and Average Number of Active Centres per Rat*

| Group No. | Treatment | No. of rats surviving longer than 4 weeks | Average progesterone in mg. absorbed per rat per 30 days | Percentage of rats developing mammary tumours in 28 weeks | Average latent period in days for appearance of first mammary tumour | Percentages of rats with multiple* mammary tumours of those developing mammary growths | Average No. of active centres per rat of those with multiple* mammary tumours |
|---|---|---|---|---|---|---|---|
| 1 | DMBA | 21/22 | — | {43·0 | {113 | 37·0 | 2·0 |
| 2 | DMBA + P ∞ | 21/23 | 87 | | | 70·0 | 5·1 |
| 3 | DMBA + P + 15 | 19/20 | 90 | 84·0 | 94 | 88·0 | 4·9 |
| 4 | DMBA + P–2 | 14/20 | 89 | 64·0 | 69 | 78·0 | 3·9 |
| 5 | P–2 | 17/20 | 92 | 0·0 | — | — | — |

* Two or more tumours per rat and fed commercial rat pellets, supplemented with greens occasionally, milk twice a week and water *ad libitum*. At 50 days of age each rat in groups of 1–4 was fed intragastrically a single 30 mg. dose of DMBA dissolved in 2 ml. of corn oil. In addition, groups 2–4 received progesterone implants. In group 2 (DMBA + P ∞), only rats bearing measurable mammary tumours (1 cm. or more in longest diameter) were given progesterone, while in group 3 (DMBA + *P* + 15) and group 4 (DMBA + *P* — 2) all rats received progesterone, beginning on the 65th and 48th day of age, respectively. Rats in control group 5 (*P* — 2) received progesterone only, beginning on the 48th day of age. The circular hormone implants (1 cm. in diameter) were made by compressing approximately 200 mg. of progesterone powder under 5–6 tons pressure. Each pellet was accurately weighed and implanted intraperitoneally for 30 days. The pellets were removed every 30 days and new pellets implanted; this procedure was continued for the duration of the experiments ($6\frac{1}{2}$ months). The fibrous capsule was stripped off each pellet after removal, the progesterone allowed to dry and then reweighed to ascertain the amount absorbed per rat per 30 days.

Beginning 4 weeks after DMBA administration, all rats were palpated weekly and the presence of any mammary tumours recorded. When the neoplasms reached 1 cm. or more in longest diameter, they were measured with callipers in two diameters, one in the longest axis of the growth and the other at right angles to it. The arithmetical mean of these two measurements was used as the measure of tumour size, and this value was graphed so that the growth progress of each neoplasm could be followed.

Portions of each tumour removed at biopsy or autopsy were fixed in 10% buffered formalin and embedded in paraffin. Sections were cut at 5 $\mu$ and stained with haematoxylin and eosin and alcian blue. Frozen sections were also cut from some tumours and stained for fat by Sudan III.

### RESULTS

Within the first 4 weeks of the experiments one or more rats died in each of the five groups from one of several causes, including suppurative bronchopneumonia, ether anaesthesia overdose, or the toxic effects of DMBA, most deaths occurring in group 4 (Table I). As no mammary tumour was observed to develop during this period, all figures and statistics have been based on the total number of rats which survived longer than four weeks.

TABLE II.—*Average Number of Active Centres per Rat in Each Group and its Standard Deviation*

| Group No. | Total No. of rats | Average No. of active centres $\bar{x}$ | Standard error of $\bar{x}$* |
|---|---|---|---|
| 1 | 21 | 0·52 | 0·16 |
| 2 | 21 | 1·86 | 0·63 |
| 3 | 19 | 3·74 | 0·67 |
| 4 | 14 | 2·07 | 0·78 |
| Weighted average 3 & 4 | 33 | 3·03 | 0·50 |
| Weighted average 2, 3 & 4 | 54 | 2·57 | 0·39 |

* Standard errors quoted are based on the original figures, but since these figures gave variance estimates which indicated heterogeneity, the data were transformed in such a manner as to reduce the heterogeneity before any comparisons were made.

### *Tumour incidence*

The average amount of progesterone absorbed per rat per 30 days in groups 2–5 was similar (Table I), and by inspection the slight difference was not significant. However, while mammary neoplasia did not arise in any animal of group 5, which received only progesterone, breast tumours developed in some rats in each of the other groups (1–4). Further, the tumour incidence was considerably increased in those animals which had received progesterone either just before, or 15 days after, DMBA administration (groups 3 and 4), compared with the combined average incidence in those which had received only DMBA (groups 1 and 2), (Table I). The tumour incidences in the latter two groups were combined and averaged, since progesterone was not administered to any rat in group 2 until it had developed a growth of measurable size (1 cm. or more in longest diameter).

An overall $\chi^2$ test on the observed numbers of rats developing tumours in groups 1–4 indicated that progesterone significantly modified the effect of DMBA ($\chi^2 = 9{\cdot}8$ for 3 d.f.). There was no significant difference in the incidence in groups 1 and 2, nor did group 3 differ significantly from group 4; however the combined incidence rate in groups 3 and 4 was significantly greater than that in groups 1 and 2*.

*Latent period*

The average time before the first tumour was detected in groups 3 and 4 was slightly shorter than the combined (refer Tumour Incidence) average observed in groups 1 and 2 (Table I). Statistically, the average of 113 days (groups 1 and 2) did not differ significantly from the average for group 3, but was significantly greater than the average for group 4. The average latent period for group 3 did not differ significantly from that of group 4, but if these two groups are pooled, the weighted average of 85 days is significantly less than the pooled average from groups 1 and 2*. This is illustrated in Fig. 1, where it can be seen that 58% and 57% of

FIG. 1.—Histogram showing increased tumour incidence and shortening of induction time in groups 3 and 4 following progesterone administration in addition to DMBA, compared with that for groups 1 and 2 which received DMBA only. Percentages in groups 1 and 2 were based on 42 rats, and in groups 3 and 4 on 19 and 14 animals, respectively.

tumours in groups 3 and 4, respectively, were first detected between 40 and 80 days after DMBA administration, while in this same period only 12% of mammary growths developed in rats of groups 1 and 2, the majority in these latter two groups first occurring between 120 and 160 days after application of the carcinogen.

*Tumour multiplicity*

More than one mammary growth arose in many animals in groups 1–4, and administration of progesterone appeared to enhance considerably multi-tumour development regardless of whether the hormone implantations were begun before or after the first neoplasm had appeared (Table I).

* Details of the statistical analyses made in each section have been omitted, but H. Scheffé's method (*Biometrika*, 1953, **40**, 87–104) was used whenever multiple comparisons were made within a set of results.

With the exception of the comparisons between groups 3 and 4, $\chi^2$ tests were made against a one-sided alternative that progesterone enhanced multi-tumour development, and were based only on rats which had developed at least one tumour*. These showed that progesterone significantly increased the probability of tumour multiplicity, but that the incidence of multiple tumours induced in rats of groups 2–4 did not differ significantly amongst themselves.

*Active centres*

The total number of multiple tumours (active centres) developing in any rat differed from animal to animal even within the same group. However, progesterone, regardless of the time of its administration, appeared to increase the average number of active centres per rat in groups 2–4 compared with the average number per rat in the control group 1 (Table I), and this difference was found to be statistically significant.

The statistical analysis of the four treatments (groups 1–4) was based on the results observed in all rats, including animals with no tumours and those with only one neoplasm (Table II). The same one-sided alternative was used for all comparisons (refer Tumour Multiplicity), except for that between groups 3 and 4. The analysis* showed that the number of active centres developing in either group 1 or group 2 was significantly lower than that in group 3, but was not significantly lower than the number in group 4, and that DMBA alone (group 1) induced significantly fewer active centres than the weighted average of the active centres in groups 2–4. Statistical analysis of the differences between the three progesterone-treated groups (2–4) revealed that while the differences in the number of active centres between groups 2 and 4 and 3 and 4 were not significant, progesterone given 15 days after DMBA administration (group 3) significantly increased the number of active centres as compared with group 2.

* See footnote page 421.

---

EXPLANATION OF PLATES

FIG. 2.—Portion of a solid type of carcinoma, showing actively proliferating epithelial cells arranged in dense cellular sheets; many of these cells contain mucin or fat vacuoles in their cytoplasm. H. & E. × 100.

FIG. 3.—Portion of a solid type of carcinoma, showing accumulation of mucin intercellularly (bottom), and the presence of several small glandular structures, some of which appear to have invaded a fibrous septum. H. & E. × 100.

FIG. 4.—Portion of a papillary cystadenocarcinoma, showing two cystic spaces separated by fibrous trabeculae and each almost entirely filled by proliferating epithelial cells which are arranged in a papillary pattern. H. & E. × 40.

FIG. 5.—Portion of a cystic space in a papillary cystadenocarcinoma, showing the presence of glandular structures and relatively scanty and fibrillary stroma within papillae. H. & E. × 100.

FIG. 6.—Papillae within a cystic space of a papillary cystadenocarcinoma, showing considerable mucinous cytoplasmic vacuolation in many of the epithelial cells, accumulation of mucin intercellularly, and the presence of several glandular structures. Alcian blue. × 100.

FIG. 7.—Portion of an adenoma, showing the nodular arrangement of numerous ductal and acinar structures, each nodule being bounded by narrow fibrous septa. H. & E. × 40.

FIG. 8.—Portion of an adenoma, showing the presence of numerous ducts and acini within a nodule, the acini containing fat droplets and eosinophilic material in their lumina, and cytoplasmic fat vacuoles in many of the epithelial cells lining both types of adenomatous structures. H. & E. × 100.

FIG. 9.—Portion of a fibroadenoma, showing numerous small solid or luminated ducts surrounded by abundant dense fibrous stroma. H. & E. × 40.

Jabara.

6

7

8

9

Jabara.

*Growth behaviour of tumours*

Three main types of tumour growth behaviour occurred in all four groups of animals (Table III). Many neoplasms grew continuously, either rapidly or gradually, until they were either removed surgically or the host was killed at the end of the experiment. This was designated " continuous growth ". Other tumours, following an initial short growth period, remained about the same size for the remainder of the experiment, and this type of growth was classified as " static ". Still other neoplasms initially showed a short period of growth and then gradually diminished in size until they either completely disappeared or were considerably smaller than 1 cm. in longest diameter. This type of progress was classified as " regressing ". Some tumours in all four groups showed a more complicated growth behaviour, involving combinations of any two of the three main types, and neoplasms exhibiting such combinations were for simplicity classified on their initial growth progress (Table III). The growth behaviour for a small percentage of tumours arising in groups 2–4 (Table III) could not be classified because most of these neoplasms arose late in the experiment and were either too small to be measured or had not been measured for a sufficient length of time to determine their true growth pattern before the hosts were killed. A few growths were undetected until the host was autopsied. Progesterone did not appear to influence tumour growth behaviour (Table III). This was confirmed statistically, when an overall test of independence of treatments and growth behaviour of tumours was not found to be significant, nor was there any marked deviation in any single observed result from that which would be expected if the hypothesis of independence were correct.

TABLE III.—*Growth Behaviour of Induced Mammary Tumours*

| Group No. | Total No. of rats with tumours | Total No. of tumours | Percentage of tumours CG | S | R | Unclassified |
|---|---|---|---|---|---|---|
| 1 | 8 | 11 | 46 | 27 | 27 | 0 |
| 2 | 10 | 39 | 56 | 26 | 8 | 10 |
| 3 | 16 | 71 | 42 | 30 | 20 | 8 |
| 4 | 9 | 29 | 31 | 38 | 21 | 10 |

CG = Continuous growth.
S = Static growth.
R = Regressing.

Microscopic examination of the neoplasms revealed no direct correlation between growth behaviour and histological picture, and a carcinoma, fibroadenoma or adenoma might all show continuous growth, or might remain static. However, while the majority of carcinomata and adenomata exhibited continuous growth, the majority of fibroadenomata remained static.

*Locations of tumours*

In all four groups there was no apparent predilection for tumours to develop on one side rather than the other, both sides being equally affected. The majority of growths in all groups arose in the first three pairs of glands, and less frequently the 4th, 5th and 6th pairs of glands were affected in descending order of frequency.

*Types of tumours*

Three main tumour types were distinguished (Table IV), and all of these occurred in each of the four groups of rats (1–4), progesterone appearing not to influence either the macroscopic or microscopic appearances of the developing growths.

TABLE IV.—*Percentage of Histologically Similar Tumour Types in Each Group*

| Group No. | Total No. of tumours | Percentage Carcinoma | Adenoma | Fibro-adenoma | Unclassi-fiable* |
|---|---|---|---|---|---|
| 1 | 11 | 73 | 0 | 9 | 18 |
| 2 | 39 | 79 | 8 | 5 | 8 |
| 3 | 71 | 56 | 20 | 12 | 12 |
| 4 | 29 | 66 | 14 | 17 | 3 |

* Tumours were unclassifiable due to complete regression before autopsy or because they were lost during paraffin embedding.

(*a*) *Carcinoma.*—The majority of neoplasms in all four groups were of this type (Table IV). Macroscopically they appeared as ovoid, partially encapsulated growths of soft to firm consistency, which weighed up to 70 g. and measured up to 6·0 × 5·5 cm. The cut surfaces revealed pink, white or cream-coloured nodular tissue which was sometimes solid, but more frequently contained multiple cysts, ranging from 1–7 mm. in diameter and filled with dark brown-coloured semi-gelatinous material; the tumours sometimes contained a necrotic focus centrally.

Microscopically carcinomata were either of a solid and poorly differentiated type, or else, and this involved the majority of growths, well-differentiated papillary cystadenocarcinomata. Most carcinomata showed both patterns and were classified on their predominant feature. The poorly differentiated type consisted predominantly of solid sheets, clumps and cords of actively proliferating, pleomorphic, hyperchromatic epithelial cells (Fig. 2), although small, or sometimes cystic, adenomatous formations of acinar or ductal type were often seen (Fig. 3). The papillary cystadenocarcinomata, on the other hand, were composed predominantly of large cystic spaces which were completely or partially filled by proliferating epithelial cells which were generally arranged in a papillary pattern (Fig. 4); mitotic figures were less frequent than in the solid type. Within these papillae the intercellular stroma was scanty and fibrillary, but ductal and acinar structures were common (Fig. 5).

In both types of carcinomata the epithelial cells frequently contained cytoplasmic mucinous vacuoles (alcian blue positive) or, less commonly, fat vacuoles (Sudan III positive) (Fig. 2 and 6); intercellular pools of mucin were also seen (Fig. 3 and 6). The stroma in both tumour types was generally relatively sparse and commonly infiltrated by large and small mononuclear cells, eosinophils and mast cells. Small or larger necrotic foci were sometimes observed, especially near the tumour centre. Myoepithelial cells were observed scattered around many of the glandular structures and also mixed with epithelial cells within the cellular sheets and clumps. Metastases were not found in any case.

(*b*) *Adenoma.*—This type of tumour was considerably less frequent than carcinoma, but appeared with approximately the same incidence as fibroadenoma (Table IV). Macroscopically they appeared as ovoid, encapsulated masses of soft consistency, weighing up to 36 g. and measuring up to 5·0 × 4·0 cm. In cross section, they were composed of cream-coloured nodules which contained multiple small cysts, ranging from less than 1–7 mm. in diameter and filled with dark brown-coloured semi-gelatinous material.

Histologically, adenomata consisted of numerous glandular structures arranged in a nodular pattern, each nodule being bounded by relatively narrow fibrous septa which contained small and large mononoclear cells, eosinophils and mast cells scattered through them (Fig. 7). Commonly the adenomatous formations consisted exclusively or predominantly of small ducts, although many tumours, in addition, contained small or cystic acinar structures. Most of the epithelial cells contained cytoplasmic fat vacuoles (Fig. 8), and mitotic figures were infrequent. In most ducts and acini myoepithelial cells were visible lying just within the basement membranes.

(c) *Fibroadenoma.*—Macroscopically these tumours appeared as encapsulated, ovoid or discoid masses of rubbery consistency, which measured up to 2·0 × 1·5 cm. and weighed up to 3 g. The cut surfaces revealed small buff-coloured or greyish-cream-coloured nodules scattered in dense white tissue.

Histologically fibroadenomata consisted of dense fibrous tissue in which were situated small solid or luminated ducts (Fig. 9) and, less frequently, small acinar formations. Both acinar and ductal epithelial cells often contained cytoplasmic fat vacuoles, and mitotic figures were rare. Myoepithelial cells could sometimes be distinguished in the lining of ducts and acini.

### DISCUSSION

The observation that progesterone *per se* did not induce mammary neoplasia in any rat of group 5 confirms the findings of Gruenstein *et al.* (1964), Huggins *et al.* (1959) and Poel (1965), who administered this hormone, respectively, to Wistar and Sprague-Dawley rats and to mice.

The reported incidences and induction times of mammary tumours and the average number of active centres developed per animal following the feeding of a single dose of DMBA to Sprague-Dawley rats have varied considerably, even when a dose of comparable size was administered (Table V). Comparison of the findings

TABLE V.—*Reported Results of Feeding DMBA to Sprague-Dawley Rats*

| Investigators | Single intra-gastric dose (mg.) | Percentage tumour incidence | Range of tumour induction time (days) | Average No. of active centres per rat |
|---|---|---|---|---|
| Huggins *et al.* (1961) | 20 | 100 | 28–60 | 6·8 |
| Huggins and Yang (1962) | 20 | 100 | 21–60 | 2·1 |
| | | | 28–39* | 5·0* |
| Huggins *et al.* (1962) | 20 | 100 | 27–72 | 2·7 |
| | | | 24–49* | 3·6* |
| Sydnor *et al.* (1962) | 20 | 100 | 25–54 | 3·8 |
| Talwalker *et al.* (1964) | 20 | 100 | 52–110 | 3·9 |
| McCormick and Moon (1965) | 20 | 100 | 46–137 | 4·95 |
| | | | 24–39† | 3·07† |
| Teller *et al.* (1966) | 20 | 70·6 | 91–218 | 5·1 |
| Young and Cowan (1963) | 50 | 62 | 50–100 | — |
| Young *et al.* (1963) | 50 | 57 | — | — |

* Results following progesterone administration from day 15 to day 45 after feeding DMBA.
† Results following induction of pregnancy from day 16 to day 29 after feeding DMBA.

in the control animals (group 1) with previously reported observations revealed that the mammary tumour incidence was slightly lower, while the range of induction time and the average number of active tumour centres per rat were both comparable with those recorded by several investigators (Table V). It appears possible that the results obtained may depend upon the substrain of Sprague-Dawley rat used.

Progesterone significantly increased the tumour incidence in the present series, and further, it did so regardless of whether it were given just before, or 15 days after, DMBA application. Huggins and his colleagues and McCormick and Moon, however, obtained a 100% tumour incidence whether or not progesterone was administered, or pregnancy induced, in addition to DMBA (Table V).

The significant decrease in tumour induction time observed in rats of group 3, which received progesterone continuously beginning 15 days after DMBA administration, confirms the observations of Huggins and co-workers (Table V). Further it was found that progesterone given two days before DMBA was just as effective in shortening the latent period. McCormick and Moon (1965) found that pregnancy induced 16–29 days after DMBA administration had a similar shortening effect on tumour induction time (Table V).

The increases in tumour multiplicity and the number of active centres per rat following progesterone administration 15 days after DMBA confirms the findings of Huggins and co-workers (Table V). However, McCormick and Moon (1965) found that pregnancy occurring 16–29 days after DMBA administration did not increase the number of tumours per rat (Table V), although further new growths arose resulting in an increase in the number of active centres per animal when the rats were mated after the first neoplasms had developed. A similar finding was obtained in group 2 of the present series following the implantation of progesterone in tumour-bearing rats. Further, it was found that when progesterone was given before DMBA there was a significant increase in the number of tumour centres developed. Thus, the average number of active centres per rat was increased in all groups receiving progesterone regardless of when the hormone was administered, but the greatest increase in number was observed in rats which received progesterone 15 days after DMBA. However, there was a high casualty rate in group 4 early in the experiment, and therefore the results in this group may not describe accurately the influence of progesterone when given before DMBA.

The observed variation in growth behaviour of DMBA-induced mammary tumours even in the same group of animals, or in the same rat, confirms the work of Young, Cowan and Sutherland (1963), Young and Cowan (1963) and Teller *et al.* (1966), and further, progesterone did not appear to influence these neoplastic growth patterns. However, Huggins *et al.* (1962) and Huggins and Yang (1962) reported that progesterone significantly enhanced the growth rate of DMBA-induced mammary tumours, and similarly McCormick and Moon (1965) observed that the tumours grew more rapidly during pregnancy, regardless of whether the rats were mated before or after mammary neoplasms had appeared.

In the present series, administration of progesterone in addition to DMBA did not modify the locations or the macroscopic or microscopic appearances of the induced tumours, regardless of when the hormone was implanted. It has been suggested that the hormonal conditions prevailing in an animal at the time when a carcinogen is exerting its effect determine the type of tumour which develops (Shay, Harris and Gruenstein, 1952; Daniel and Prichard, 1964). However, this does not appear to be the complete answer in view of the fact that similar histological tumour types were observed in all four groups of rats of the present experiments, despite the different hormonal conditions which existed in each group. The absence of adenomata from group 1 (Table IV) was not considered to be significant as so few tumours developed in this group within the duration of the experiment ($6\frac{1}{2}$ months). This tentative conclusion was subsequently proved correct when this group of rats was kept alive for a further $2\frac{1}{2}$ months after the conclusion of the main experiment. By the end of 9 months the number of rats in this group bearing tumours had increased from 8 to 17, and the number of mammary neoplasms had increased from 11 to 56, of which 13 (23%) were adenomata.

While myoepithelial cells were invariably present in the growths, they appeared to make little contribution to the tumours, and in all growths of the present series, the epithelial cell was the predominant cell type, regardless of whether the growths were benign or malignant and whether or not progesterone had also been administered.

Thus, while progesterone *per se* was not carcinogenic in the Sprague-Dawley rat, it was found that the hormone significantly enhanced mammary tumorgenesis induced by DMBA, regardless of whether the steroid was administered 2 days before feeding the carcinogen, or 15 days after DMBA application (confirming the findings of Huggins *et al.*, 1962), or after the first mammary growths had appeared. From this last observation, it was concluded that exogenous progesterone was as effective in enhancing DMBA-induced tumorgenesis, as McCormick and Moon (1965) found the induction of pregnancy to be in tumour-bearing rats.

The mechanism by which progesterone enhances the tumorgenic response is not yet known. However, the work of several authors suggests that the anterior pituitary hormones, prolactin and somatotrophin (STH), maybe of prime importance in the induction of mammary growths in rats by DMBA or 3MCA (Young, 1961; Sterental *et al.*, 1963; Talwalker, Meites and Mizuno, 1964), and there is some evidence to suggest that progesterone may act indirectly via the pituitary gland and hypothalamus inducing an increased secretion of prolactin and possibly STH as well (Rothchild, 1960; Huggins, Mainzer and Briziarelli, 1958). Bock and Dao (1961) have postulated that the end effects of hormones upon tumour formation may be due primarily to an alteration of the target cells, rather than to changes in the amount of carcinogen to which they are exposed. The findings in group 2 of the present series appear to support this view, progesterone apparently enhancing the appearance of many tumours which might otherwise have remained dormant. Bock and Dao (1961) suggested that the alteration possibly may effect either the absorption of DMBA by the mammary epithelial cells from the storage depot of carcinogen in the mammary adipose tissue, or the metabolism of DMBA within the cells. The former suggestion is currently being investigated in this laboratory by means of tritiated DMBA and autoradiography.

### SUMMARY

One hundred and five non-inbred Sprague-Dawley virgin female rats were divided into 5 groups. Rats in groups 1–4 were given a single intragastric dose of 30 mg. of DMBA at 50 days of age. Group 1 received no other treatment. Groups 2, 3 and 4 received in addition a 200 mg. i.p. implant of progesterone every 30 days. The time of administration of the first dose of progesterone differed in each group: in group 2 it was given after the appearance of the first mammary tumour, in group 3, 15 days after DMBA administration and in group 4, two days before the dose of DMBA. Animals in group 5 received progesterone only in the same dosage, the first implant being given at 48 days of age. The experiments were continued for 6½ months.

Mammary tumours developed in groups 1–4, but none arose in any animal in group 5. Progesterone significantly increased the incidence of mammary neoplasms and reduced the time for tumour induction in groups 3 and 4, administration of the hormone just before DMBA application being as effective in this regard as when it was given 15 days after DMBA. Progesterone also significantly enhanced the development of multiple tumours and increased the number of neoplasms per rat, regardless of whether the hormone implantations were begun before or after tumour appearance. The increase in the number of active tumour centres per rat was greatest when progesterone was administered 15 days after DMBA (group 3), but as a relatively high casualty rate cocurred in group 4 early in the experiment the results in this group may not describe accurately the influence of progesterone administration starting two days before DMBA.

Progesterone did not appear to influence the side or site of neoplastic development, nor the growth behaviour or macroscopic and microscopic appearances of the tumours.

This work was carried out during the tenure of a Research Training Grant from the World Health Organization, Geneva.

The author wishes to thank Professor E. Cotchin, Department of Pathology, Royal Veterinary College, London, for his support and encouragement and the many helpful discussions during the course of these experiments, and Miss A. Doig, Department of Statistics, University of Melbourne, for carrying out the statistical analysis of the data.

### REFERENCES

BIANCIFIORI, C. AND CASCHERA, F.—(1962) 'The Morphological Precursors of Cancer', edited by L. Severi. Proceedings of an International Conference held at the University of Perugia, 26th–30th June, 1961 (Perugia, Division of Cancer Research), pp. 369–373.

BOCK, F. G. AND DAO, T. L.—(1961) *Cancer Res.*, **21**, 1024.

DANIEL, P. M. AND PRICHARD, M. M. L.—(1964) *Br. J. Cancer*, **18**, 513.

DAO, T. L., GREINER, M. AND SUNDERLAND, H.—(1959) *Proc. Am. Ass. Cancer Res.*, **3**, 14.

DAO, T. L. AND SUNDERLAND, H.—(1959) *J. natn. Cancer Inst.* **23,** 567.

GRUENSTEIN, M., SHAY, H. AND SHIMKIN, M. B.—(1964) *Cancer Res.*, **24**, 1656.

HOWELL, J. S.—(1960) *Br. J. Cancer*, **14**, 657.

HUGGINS, C., BRIZIARELLI, G. AND SUTTON, H. JR.—(1959) *J. exp. Med.*, **109**, 25.

HUGGINS, C., GRAND, L. C. AND BRILLANTES, F. P.—(1961) *Nature, Lond.*, **189**, 204.

HUGGINS, C., MAINZER, K. AND BRIZIARELLI, G.—(1958) *Recent Prog. Horm. Res.*, **14**, 77.

HUGGINS, C., MOON, R. C. AND MORII, S.—(1962) *Proc. U.S. natn. Acad. Sci.*, **48**, 379.

HUGGINS, C. AND YANG, N. C.—(1962) *Science, N.Y.*, **137**, 257.

MCCORMICK, G. M. AND MOON, R. C.—(1965) *Br. J. Cancer*, **19**, 160.

MARCHANT, J.—(1963) *Br. J. Cancer*, **17**, 119.

POEL, W. E.—(1965) *Br. J. Cancer*, **19**, 824.

ROTHCHILD, I.—(1960) *Endocrinology*, **67**, 9.

SHAY, H., HARRIS, C. AND GRUENSTEIN, M.—(1952) *J. natn. Cancer Inst.*, **13**, 307.
STERENTAL, A., DOMINGUEZ, J. M., WEISSMAN, C. AND PEARSON, O. H.—(1963) *Cancer Res.*, **23**, 481.
SYDNOR, K. L., BUTENANDT, O., BRILLANTES, F. P. AND HUGGINS, C.—(1962) *J. natn. Cancer Inst.*, **29**, 805.
TALWALKER, P. K., MEITES, J. AND MIZUNO, H.—(1964) *Proc. Soc. exp. Biol. Med.*, **116**, 531.
TELLER, M. N., STOCK, C. C., STOHR, G., MERKER, P. C., KAUFMAN, R. J., ESCHER, G. C. AND BOWIE, M.—(1966) *Cancer Res.*, **26**, 245.
YOUNG, S.—(1961) *Nature, Lond.*, **190**, 356.
YOUNG, S. AND COWAN, D. M.—(1963) *Br. J. Cancer*, **17**, 85.
YOUNG, S., COWAN, D. M. AND SUTHERLAND, L. E.—(1963) *J. Path. Bact.*, **85**, 331.